# Supplementary material for: Mississippi Farmers’ Interest in and Experience with Farm to School
Source: Int J Environ Res Public Health. 2022 Jun 30;19(13):8025. doi: 10.3390/ijerph19138025 (PMC9265993; doi:10.3390/ijerph19138025)
Supplement: Supplementary file 1 [file ijerph-19-08025-s001.zip › ijerph-1763693-supplementary.pdf]

Farm to School -- Farmer Survey

You are invited to participate in a web-based online survey about Mississippi small farmers, their farm products, and their interest in Farm to School activities. This is a research project being conducted by Jessica Thomson, a researcher with the USDA ARS Stoneville Research Center.

The survey should take about 10-15 minutes to complete.

PARTICIPATION
Your participation in this survey is voluntary. You may refuse to take part in the research or exit the survey at any time without penalty. You are free to decline to answer any question you do not wish to answer for any reason.

If you participate in the survey, you will receive a $20 gift card for your time.

BENEFITS
You will receive no direct benefits from participating in this research study. However, your responses may help us learn more about small farmers, their products, and their interest or involvement in Farm to School activities in Mississippi.

RISKS
There are no foreseeable risks involved in participating in this study other than those encountered in day-to-day life.

CONFIDENTIALITY
Your information will be assigned an identification (ID) number that is unique to this study. The list connecting your name to this number will be kept on an encrypted USB flash drive that only the Principal Investigator will be able to access. Your survey answers will be sent to a secure, encrypted online web server that is password protected. No names or identifying information will be included in any publications or presentations based on the data collected. Your responses to this survey will remain confidential.

CONTACT

If you have questions about the study, please contact the Principal Investigator, Dr. Jessica Thomson via phone 225-892-3662 or via email jessica.thomson@usda.gov. If you have any questions about your rights as a research participant, please contact Delta State University Institutional Review Board at Kent Wyatt Hall Room 239, Cleveland, MS via phone 662-846-4700 or email irb@deltastate.edu.

ELECTRONIC CONSENT

You may print a copy of the consent form for your records. Clicking on the "Consent" button indicates that:

1. You have read the information above.
2. You voluntarily agree to participate.
3. You are 18 years of age or older.

Q1 Click below to begin the survey. In doing so, you agree to participate in the survey and to the use of your survey responses as described previously. If you do not wish to participate in the survey, please close your browser.

- [ ] Consent

First, let's make sure you fit the USDA definition of a small farm.

Q2 USDA defines a small farm as an operation with gross cash farm income under $250,000. Does your farm or business fit the definition?

- ☐ Yes
- ☐ No -- farm gross cash income is greater than $250,000
- ☐ No -- I don't own or operate a farm Go to Q13

Now let's gather some information about your farm.

Q3 What is the name of your farm or business?

Q4 In what town is your farm or business located?

Q5 Approximately, how many acres is your farm?

Q6 Does your farm have any certifications? Please select all that apply.

- [ ] Fairtrade
- [ ] GAP - Good Agricultural Practices
- [ ] GHP - Good Handling Practices
- [ ] Humane
- [ ] Naturally grown
- [ ] Non-GMO
- [ ] Organic
- [ ] Other
- [ ] None

If other, please specify certification(s).

Q7 Do you use the Internet to do any of the following? Please select all that apply.

- ☐ Purchase supplies, equipment or other materials for farm operation
- ☐ Use online or peer learning farm resources (webinars, tutorials, peer user groups, etc.)
- ☐ Use online farm business products and services (business planning, accounting, banking, etc.)
- ☐ Identify sources or submit requests/proposals for funding, grants, or subsidies
- ☐ Access price and market information
- ☐ Website for your farm or business
- ☐ Facebook page for your farm or business
- ☐ Other
- ☐ None
- ☐ Do not have internet access

If other, please specify the use(s).

[ ]

Next, we would like to know about foods you grow, raise, or produce on your farm.

Q8 Please select all fruits that are grown on your farm.

- ☐ Apples
- ☐ Blackberries
- ☐ Blueberries
- ☐ Cantaloupe
- ☐ Figs
- ☐ Grapes, bunch
- ☐ Grapes, muscadine
- ☐ Peaches
- ☐ Pears
- ☐ Plums
- ☐ Raspberries
- ☐ Strawberries
- ☐ Watermelon
- ☐ Other
- ☐ None

If other, please specify type(s) of fruit.

Q9 Please select all vegetables that are grown on your farm.

- ☐ Bell peppers (green or colored)
- ☐ Black eyed peas
- ☐ Broccoli
- ☐ Butter beans
- ☐ Cabbage
- ☐ Carrots
- ☐ Cauliflower
- ☐ Cucumbers
- ☐ Eggplant
- ☐ Green beans
- ☐ Green peas
- ☐ Greens (collard, mustard, turnip)
- ☐ Kale
- ☐ Kohlrabi
- ☐ Leeks
- ☐ Mushrooms
- ☐ Okra
- ☐ Onions
- ☐ Purple hull peas
- ☐ Radishes
- ☐ Sweet corn
- ☐ Sweet potatoes
- ☐ Swiss chard
- ☐ Tomatoes
- ☐ White potatoes
- ☐ Yellow squash
- ☐ Zucchini
- ☐ Other
- ☐ None

If other, please specify type(s) of vegetables.

Q10 Please select all other products that are grown, raised, or produced on your farm.

- ☐ Beef
- ☐ Chicken
- ☐ Cow's milk
- ☐ Eggs
- ☐ Herbs
- ☐ Honey
- ☐ Pecans
- ☐ Pork
- ☐ Other
- ☐ None

If other, please specify type(s) of products.

Q11 Please select the harvest month(s) for your farm products. More than one month may be selected.

	Jan	Feb	Mar	Apr	May	Jun	Jul	Aug	Sep	Oct	Nov	Dec
Apples	☐	☐	☐	☐	☐	☐	☐	☐	☐	☐	☐	☐
Blackberries	☐	☐	☐	☐	☐	☐	☐	☐	☐	☐	☐	☐
Blueberries	☐	☐	☐	☐	☐	☐	☐	☐	☐	☐	☐	☐
Cantaloupe	☐	☐	☐	☐	☐	☐	☐	☐	☐	☐	☐	☐
Figs	☐	☐	☐	☐	☐	☐	☐	☐	☐	☐	☐	☐
Grapes, bunch	☐	☐	☐	☐	☐	☐	☐	☐	☐	☐	☐	☐
Grapes, muscadine	☐	☐	☐	☐	☐	☐	☐	☐	☐	☐	☐	☐
Peaches	☐	☐	☐	☐	☐	☐	☐	☐	☐	☐	☐	☐
Pears	☐	☐	☐	☐	☐	☐	☐	☐	☐	☐	☐	☐
Plums	☐	☐	☐	☐	☐	☐	☐	☐	☐	☐	☐	☐
Raspberries	☐	☐	☐	☐	☐	☐	☐	☐	☐	☐	☐	☐
Strawberries	☐	☐	☐	☐	☐	☐	☐	☐	☐	☐	☐	☐
Watermelon	☐	☐	☐	☐	☐	☐	☐	☐	☐	☐	☐	☐
Bell peppers	☐	☐	☐	☐	☐	☐	☐	☐	☐	☐	☐	☐
Black eyed peas	☐	☐	☐	☐	☐	☐	☐	☐	☐	☐	☐	☐
Broccoli	☐	☐	☐	☐	☐	☐	☐	☐	☐	☐	☐	☐
Butter beans	☐	☐	☐	☐	☐	☐	☐	☐	☐	☐	☐	☐
Cabbage	☐	☐	☐	☐	☐	☐	☐	☐	☐	☐	☐	☐
Carrots	☐	☐	☐	☐	☐	☐	☐	☐	☐	☐	☐	☐
Cauliflower	☐	☐	☐	☐	☐	☐	☐	☐	☐	☐	☐	☐
Cucumbers	☐	☐	☐	☐	☐	☐	☐	☐	☐	☐	☐	☐
Eggplant	☐	☐	☐	☐	☐	☐	☐	☐	☐	☐	☐	☐
Green beans	☐	☐	☐	☐	☐	☐	☐	☐	☐	☐	☐	☐
Green peas	☐	☐	☐	☐	☐	☐	☐	☐	☐	☐	☐	☐
Greens	☐	☐	☐	☐	☐	☐	☐	☐	☐	☐	☐	☐
Kale	☐	☐	☐	☐	☐	☐	☐	☐	☐	☐	☐	☐
Kohlrabi	☐	☐	☐	☐	☐	☐	☐	☐	☐	☐	☐	☐
Leeks	☐	☐	☐	☐	☐	☐	☐	☐	☐	☐	☐	☐
Mushrooms	☐	☐	☐	☐	☐	☐	☐	☐	☐	☐	☐	☐
Okra	☐	☐	☐	☐	☐	☐	☐	☐	☐	☐	☐	☐
Onions	☐	☐	☐	☐	☐	☐	☐	☐	☐	☐	☐	☐
Purple hull peas	☐	☐	☐	☐	☐	☐	☐	☐	☐	☐	☐	☐
Radishes	☐	☐	☐	☐	☐	☐	☐	☐	☐	☐	☐	☐
Sweet corn	☐	☐	☐	☐	☐	☐	☐	☐	☐	☐	☐	☐

Sweet potatoes	☐	☐	☐	☐	☐	☐	☐	☐	☐	☐	☐	☐
Swiss chard	☐	☐	☐	☐	☐	☐	☐	☐	☐	☐	☐	☐
Tomatoes	☐	☐	☐	☐	☐	☐	☐	☐	☐	☐	☐	☐
White potatoes	☐	☐	☐	☐	☐	☐	☐	☐	☐	☐	☐	☐
Yellow squash	☐	☐	☐	☐	☐	☐	☐	☐	☐	☐	☐	☐
Zucchini	☐	☐	☐	☐	☐	☐	☐	☐	☐	☐	☐	☐
Beef	☐	☐	☐	☐	☐	☐	☐	☐	☐	☐	☐	☐
Chicken	☐	☐	☐	☐	☐	☐	☐	☐	☐	☐	☐	☐
Cow's milk	☐	☐	☐	☐	☐	☐	☐	☐	☐	☐	☐	☐
Eggs	☐	☐	☐	☐	☐	☐	☐	☐	☐	☐	☐	☐
Herbs	☐	☐	☐	☐	☐	☐	☐	☐	☐	☐	☐	☐
Honey	☐	☐	☐	☐	☐	☐	☐	☐	☐	☐	☐	☐
Pecans	☐	☐	☐	☐	☐	☐	☐	☐	☐	☐	☐	☐
Pork	☐	☐	☐	☐	☐	☐	☐	☐	☐	☐	☐	☐

Q12 Please select the amount (in pounds) of the farm products you grow, raise or produce. Animal weight is based on "live on hoof" weight.

	1-50	51-100	101-200	201-500	501-1000	1001+
Apples	☐	☐	☐	☐	☐	☐
Blackberries	☐	☐	☐	☐	☐	☐
Blueberries	☐	☐	☐	☐	☐	☐
Cantaloupe	☐	☐	☐	☐	☐	☐
Figs	☐	☐	☐	☐	☐	☐
Grapes, bunch	☐	☐	☐	☐	☐	☐
Grapes, muscadine	☐	☐	☐	☐	☐	☐
Peaches	☐	☐	☐	☐	☐	☐
Pears	☐	☐	☐	☐	☐	☐
Plums	☐	☐	☐	☐	☐	☐
Raspberries	☐	☐	☐	☐	☐	☐
Strawberries	☐	☐	☐	☐	☐	☐
Watermelon	☐	☐	☐	☐	☐	☐
Bell peppers	☐	☐	☐	☐	☐	☐
Black eyed peas	☐	☐	☐	☐	☐	☐
Broccoli	☐	☐	☐	☐	☐	☐
Butter beans	☐	☐	☐	☐	☐	☐
Cabbage	☐	☐	☐	☐	☐	☐
Carrots	☐	☐	☐	☐	☐	☐
Cauliflower	☐	☐	☐	☐	☐	☐
Cucumbers	☐	☐	☐	☐	☐	☐
Eggplant	☐	☐	☐	☐	☐	☐
Green beans	☐	☐	☐	☐	☐	☐
Green peas	☐	☐	☐	☐	☐	☐
Greens	☐	☐	☐	☐	☐	☐
Kale	☐	☐	☐	☐	☐	☐
Kohlrabi	☐	☐	☐	☐	☐	☐
Leeks	☐	☐	☐	☐	☐	☐
Mushrooms	☐	☐	☐	☐	☐	☐
Okra	☐	☐	☐	☐	☐	☐
Onions	☐	☐	☐	☐	☐	☐
Purple hull peas	☐	☐	☐	☐	☐	☐
Radishes	☐	☐	☐	☐	☐	☐
Sweet corn	☐	☐	☐	☐	☐	☐

Sweet potatoes	☐	☐	☐	☐	☐	☐
Swiss chard	☐	☐	☐	☐	☐	☐
Tomatoes	☐	☐	☐	☐	☐	☐
White potatoes	☐	☐	☐	☐	☐	☐
Yellow squash	☐	☐	☐	☐	☐	☐
Zucchini	☐	☐	☐	☐	☐	☐
Beef	☐	☐	☐	☐	☐	☐
Chicken	☐	☐	☐	☐	☐	☐
Cow's milk	☐	☐	☐	☐	☐	☐
Eggs	☐	☐	☐	☐	☐	☐
Herbs	☐	☐	☐	☐	☐	☐
Honey	☐	☐	☐	☐	☐	☐
Pecans	☐	☐	☐	☐	☐	☐
Pork	☐	☐	☐	☐	☐	☐

Q13 Do you sell home-made food products? Please select all that apply.

- ☐ Home canned goods such as jams, jellies, salsa, relishes, etc.
- ☐ Baked goods such as bread, pies, cakes, cookies, etc.
- ☐ Other
- ☐ None

If other, please specify.

Next, we would like to know about your selling practices.

Q14 How do you sell your farm or business products? Please select all that apply.

- ☐ CSA - Community Supported Agriculture
- ☐ Commercial vendor
- ☐ Distributor
- ☐ Farmers' market
- ☐ Off farm stand/store
- ☐ On farm stand/store
- ☐ Restaurant
- ☐ School
- ☐ Other

If other, please specify.

____

Q15 Have you ever sold your farm or business products to K-12 schools in the past?

- ☐ Yes
- ☐ No

Q16 How easy or difficult was your experience selling to schools?

- ☐ Very easy
- ☐ Easy
- ☐ Neither easy nor difficult
- ☐ Difficult
- ☐ Very difficult

Please explain your ranking/selection.

Q17 What challenges stop you from selling to schools? Please select all that apply.

- ☐ I don't have a relationship with school food service staff.
- ☐ The schools I approached in the past were not interested.
- ☐ I have difficulty guarantying a specific quantity on a specific date.
- ☐ Seasonality of my produce doesn't fit with school ordering schedules.
- ☐ School's contract limits who it can purchase from.
- ☐ Schools haven't been willing to pay the price I need.
- ☐ Food safety regulations
- ☐ Delivering logistics/transportation
- ☐ Insurance liability
- ☐ Storage space
- ☐ Other

If other, please specify.

Now, we would like to know what Farm to School activities may interest you. Note that forward contracting is an agreement between a buyer and seller to purchase/sell a product at a set price at a future point in time.

Q18 I am interested in discussing the following with schools

	Strongly disagree	Somewhat disagree	Neither disagree nor agree	Somewhat agree	Strongly agree
Forward contracting	☐	☐	☐	☐	☐
Food planning for the next growing season	☐	☐	☐	☐	☐
Having visitors at my farm for field trips, teacher workshops, or student worker internships	☐	☐	☐	☐	☐
Visiting schools for agricultural education such as farmer in the classroom or taste-testing in the cafeteria	☐	☐	☐	☐	☐
Planting more acres of product to sell to schools	☐	☐	☐	☐	☐

Finally, we would like to know a bit more about you.

Q19 What is your first and last name?

Q20 Are you male or female?

- ☐ Male
- ☐ Female
- ☐ Prefer not to say

Q21 What is your age (in years)?

- ☐ 18 to 24
- ☐ 25 to 44
- ☐ 45 to 64
- ☐ 65+
- ☐ Prefer not to say

Q22 Are you Hispanic or Latino?

- ☐ Yes
- ☐ No
- ☐ Prefer not to say

Q23 What is your race? You may select more than one.

- ☐ Black or African American
- ☐ White
- ☐ American Indian or Alaska Native
- ☐ Asian
- ☐ Native Hawaiian or Pacific Islander
- ☐ Other
- ☐ Prefer not to say

It other, please specify.

[ ]

Q24 Which of the following best describes your marital status?

- ☐ Married
- ☐ Widowed
- ☐ Divorced
- ☐ Separated
- ☐ Never married
- ☐ Living with partner
- ☐ Prefer not to say

Q25 What is the highest grade or level of school you have completed or highest degree you received?

- ☐ < High school graduate
- ☐ High school graduate/GED
- ☐ Some college
- ☐ Associate, technical or vocational degree
- ☐ Bachelor's degree
- ☐ Master's degree
- ☐ Professional or doctoral degree
- ☐ Prefer not to say

You have unanswered questions about your farm products. Please use the back button to check your responses. Otherwise click next to submit your responses.

You have unanswered questions about your selling practices. Please use the back button to check your responses. Otherwise click next to submit your responses.

You have unanswered questions about your interest in Farm to School activities. Please use the back button to check your responses. Otherwise click next to submit your responses.

Q26 May we contact you about your survey responses?

- [ ] Yes
- [ ] No

Q27 What is a good telephone number for you? Please enter as xxx-xxx-xxxx.

Thank you for completing this survey.
Your responses are very important to our research!
